# Overexpression of *Myo*-Inositol Oxygenase (*TaMIOXA*) Enhances the Drought and High-Temperature Resistance of *Triticum aestivum* L.

**DOI:** 10.3390/ijms262210894

**Published:** 2025-11-10

**Authors:** Sen Zhang, Shuaitao Huang, Lanxiang Lei, Kunpu Zhang, Yuhang Liu, Pengfei Shi, Daowen Wang, Wenmei Zhou, Wenjing Qi, Zihan Zhang, Yimeng Liu, Wenming Zheng, Kun Cheng

**Affiliations:** 1 Collaborative Innovation Center of Henan Grain Crops, State Key Laboratory of Wheat and Maize Crop Science, College of Life Sciences, Henan Agricultural University, Zhengzhou 450046, Chinahuang1883783@163.com (S.H.); kpzhang@henau.edu.cn (K.Z.); dwwang@henau.edu.cn (D.W.);; 2 Xinyang Academy of Agricultural Sciences, Xinyang 464000, China; spf-0527@163.com

**Keywords:** wheat, *Myo*-inositol oxygenase, drought, high temperatures, stress resistance

## Abstract

Wheat (*Triticum aestivum* L.) is the most widely cultivated staple food crop globally. As a primary food source for 35–40% of the world’s population, the stability of its yield is directly linked to global food security. However, extreme weather events triggered by climate change have led to reductions in wheat yield, resulting in an urgent need to enhance the stress tolerance of wheat against drought and high temperatures. In this study, we successfully isolated and cloned a *myo*-inositol oxygenase gene from wheat. Further research revealed that high temperatures and drought stress significantly increased the expression level of the *TaMIOXA* gene in wheat leaves. A batch of overexpressing lines was obtained via Agrobacterium-mediated transformation. Compared to the control group, wheat plants with molecularly modified *TaMIOXA* overexpression exhibited stronger resistance to high temperatures and drought. This significantly increased their survival rates by 10% to 40%. The cumulative amount of hydrogen peroxide decreased from 7.86 × 10^−4^ to 1.54 × 10^−2^ mmol/g, and that of malondialdehyde decreased from 8.42 × 10^−7^ to 2.21 × 10^−6^ mmol/g. This confirms that overexpression of *myo*-inositol oxygenase significantly enhances wheat’s tolerance to drought and high temperatures. This study offers valuable genetic resources for wheat stress tolerance.

## 1. Introduction

Wheat (*Triticum aestivum* L.) is the most widely cultivated staple crop worldwide [1], spanning a broad latitudinal range from 60° N to 40° S and occupying 30.7% of the global grain-cropping area [2]. It serves as a primary food source for 35–40% of the world’s population [3], supplying approximately 20% of humanity’s caloric and protein requirements [4]. Consequently, the stability of wheat yields is directly linked to global food security [5]. However, extreme weather events driven by climate change are threatening wheat production systems at an unprecedented rate [6]. Current studies indicate that for every 1 °C rise in global temperature, the environmental adaptability of existing wheat varieties declines by 8.7% [7]. High temperatures directly damage the photosynthetic apparatus of wheat leaves, disrupting their chloroplast structure [8] and reducing Rubisco activity by 55–70%, which severely weakens carbon assimilation [9]. Moreover, stress-induced bursts of reactive oxygen species (ROS) compromise the cellular membrane system, trigger oxidative stress [10], and accelerate plant senescence [11]. In the water dimension, drought stress leads to abnormal stomatal opening and closing, markedly decreasing water use efficiency and thereby inhibiting nutrient uptake and material transport [12]. In addition, drought stress damages the cellular membrane system of crops [13], elevates the production of reactive oxygen species (ROS), and disrupts the balance between ROS generation and scavenging [14]. This leads to membrane lipid peroxidation and oxidative stress in plant cells, resulting in increased malondialdehyde (MDA) content and heightened cell-membrane permeability [15]. Moreover, drought stress inactivates membrane-associated proteins, enzymes, and other biomolecules, impairing the structure and function of the biofilm and, in severe cases, causing crop death [16].

Currently, research on plant resistance to abiotic stresses such as drought and high temperature has evolved into a multidimensional, multilevel systematic framework. Significant progress has been made in screening and functional analysis of related genes. For instance, TaSnRK2.10 enhances drought tolerance by phosphorylating TaERD15 and TaENO1 [2], whereas TaSHN1 improves drought resistance by modulating leaf architecture without compromising yield [17]. In terms of hormonal and signaling regulation, progesterone contributes to the stability of photosystem II under heat stress [18]. Moreover, endoplasmic reticulum stress-related miRNAs are widely implicated in stress responses [19]. In rye and maize, exogenous application of 2,4-epibrassinolide and introduction of C4-type PEPC genes have been shown to markedly enhance photosynthetic performance and improve tolerance to high temperatures [6,20]. In addition, members of transcription-factor families such as MYB and SPL (e.g., TaMYB31 and TaSPL6) exert opposite regulatory effects on drought-response processes, with MYB acting as a positive regulator and SPL as a negative regulator [21,22]. Moreover, other crop genes—including maize ZmSCE1e, ZmERF21, and tobacco NtabDOG1L—enhance drought and heat tolerance through sumoylation modification and the regulation of hormone-signaling pathways and antioxidant mechanisms, respectively [23,24,25]. The down-regulation of the cytokinin receptor SlHK2 enhances tomato resistance to combined stresses [26], while the heat shock factor TaHsfA2d also participates in the response to phosphorus deficiency [27]. These studies systematically elucidate the complex regulatory network involving multiple species and genes in abiotic stress responses, and they provide a wealth of candidate genes and a solid theoretical foundation for the genetic improvement of wheat stress resistance.

*Myo*-inositol oxygenase (*MIOX*) is a pivotal metabolic enzyme that plays a crucial role in plant growth, development, and metabolism, particularly in response to abiotic stress [28]. As a key enzyme in the inositol metabolic pathway, *MIOX* regulates the cellular inositol pool, thereby influencing the synthesis of L-ascorbic acid (L-ASA) and the biosynthesis of cell-wall polysaccharides [29]. Moreover, *MIOX* significantly contributes to plant tolerance against various abiotic stresses, including drought, salinity, and low temperature. For example, overexpression of the *MIOX* gene in cotton enhances the plant’s drought and salt resistance [30], while its overexpression in Arabidopsis improves tolerance to a range of abiotic stresses [31]. Current studies have demonstrated that MIOX is associated with plant resistance across various species. Elevating the expression level of *myo*-inositol oxygenase enhances plant tolerance to abiotic stresses. Moreover, the expression pattern of the *MIOX* gene varies among different tissues and is induced by drought, salinity, and cold stress.

In view of the increasingly severe drought and high-temperature conditions that have affected Henan in recent years [32], we aimed to investigate the role of TaMIOX in wheat tolerance to these stresses. We selected the main cultivated wheat varieties from different latitudes in the Huang-Huai-Hai Wheat Region of China for this study. The resulting transgenic lines were then subjected to RT-qPCR and leaf protein extraction, confirming that the *pCAMBIA1302-TaMIOXA* construct functions effectively in wheat. The transgenic lines overexpressing *myo*-inositol oxygenase and the corresponding control plants were subjected to simulated drought and high-temperature stress. The overexpression group displayed a markedly higher survival rate than the control group, demonstrating that elevated *TaMIOX* expression significantly enhances wheat tolerance to combined drought and heat stress. In response to the increasingly severe meteorological stress issues in the Huang-Huai-Hai wheat region, particularly in Henan Province, this study aims to provide precise genetic targets for the stress resistance genetic improvement of the main cultivated wheat varieties in this area. To furnish new genetic resources for the in-depth elucidation of the molecular mechanisms underlying wheat’s response to combined drought and high-temperature stress, and to consolidate the theoretical and experimental foundation for stress-tolerant molecular breeding in wheat.

## 2. Results

### 2.1. Gene Sequence Information of Myo-Inositol Oxygenase

**Figure 1:**
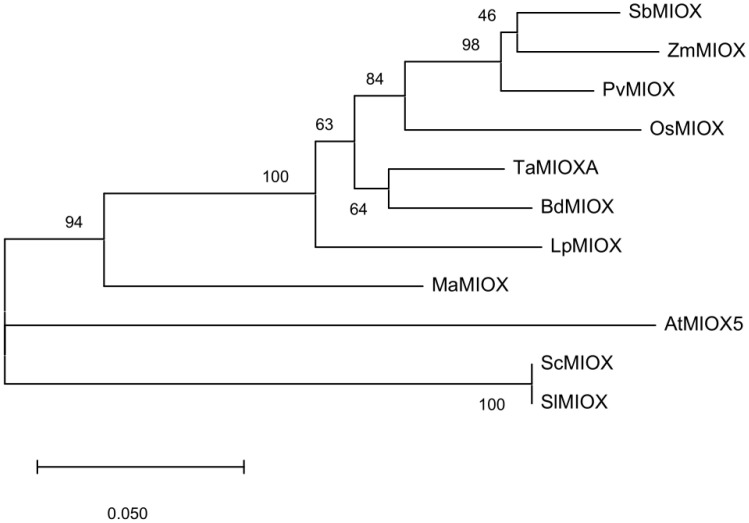
A phylogenetic tree was generated using the wheat-derived *TaMIOXA* sequence. The tree is drawn to scale, with branch lengths measured in the number of substitutions per site. The abbreviations and corresponding species names are as follows: TaMIOXA (*Triticum aestivum* L.), OsMIOX (*Oryza sativa* L.), BdMIOX (*Brachypodium distachyon* (L.) P. Beauv.), LpMIOX (*Lolium perenne* L.), PvMIOX (*Panicum virgatum* L.), SbMIOX (*Sorghum bicolor* (L.) Moench), ZmMIOX (*Zea mays* L.), AtMIOX5 (*Arabidopsis thaliana* (L.) Heynh.), MaMIOX (*Musa acuminata* Colla), ScMIOX (*Solanum chilense*), SlMIOX (*Solanum lycopersicum* L.).

The coding sequence of *TaMIOXA* obtained from wheat was aligned with the reference sequence deposited in the public database (Figure S1). One nucleotide substitution was identified across all four wheat varieties. The phylogenetic tree was constructed from retrieved coding sequences (CDSs) encoding *TaMIOXA*-homologous proteins to elucidate evolutionary relationships among these homologs (Figure 1).

### 2.2. Changes in Myo-Inositol Oxygenase Transcription Level Under Drought and High-Temperature Stress

**Figure 2:**
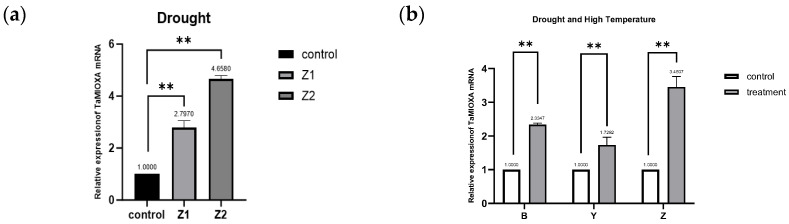
(**a**) Expression of *TaMIOXA* in Zhengmai 7698 after 5-day drought treatment. (**b**) Expression levels of *TaMIOXA* in Bainong 207, Yangmai 13, and Zhengmai 7698 following 24-h combined drought and high-temperature treatment for *MIOXA* overexpression lines. Statistical analyses were performed using one-way analysis of variance (ANOVA). ** *p* < 0.01, Data are presented as mean ± SD; all experiments included n = 3 biological replicates.

We first subjected the cultivar Zhengmai 7698 to the drought regimen and subsequently extracted total RNA from its leaves. Quantitative reverse transcription PCR (RT qPCR) analysis revealed that drought stress significantly upregulated the transcription of the *myo*-inositol oxygenase gene in Zhengmai 7698. Subsequently, two additional wheat cultivars—Yangmai 13 and Bainong 207—were subjected to 40 °C heat treatment for 24 h. Quantitative reverse transcription PCR (RT-qPCR) analysis revealed that the abundant transcript of *TaMIOX* was significantly upregulated in all three wheat varieties. This demonstrates that both drought and high-temperature stress markedly upregulated *TaMIOX* transcription in wheat leaves (Figure 2).

### 2.3. Construction of Wheat MIOXA Overexpression Lines

**Figure 3:**
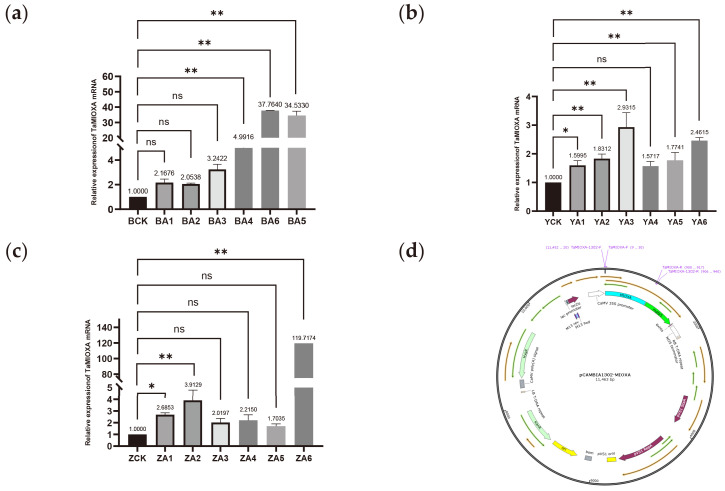
(**a**) The expression level of *TaMIOXA* in the Bainong 207 overexpression strain. (**b**) The expression level of *TaMIOXA* in the Yangmai 13 overexpression strain. (**c**) The expression level of *TaMIOXA* in the Zhengmai 7698 overexpression strain. Statistical analyses were performed using one-way analysis of variance (ANOVA). ** *p* < 0.01, * *p* < 0.05; ns, not significant. Data are presented as mean ± SD; all experiments included n = 3 biological replicates. (**d**) *pCAMBIA1302-TaMIOXA* plasmid map.

For the construction of the *pCAMBIA1302-TaMIOXA* vector plasmid map, RNA was isolated from wheat leaves, and the *TaMIOXA* coding sequence was amplified from reverse-transcribed cDNA. The *pCAMBIA1302-TaMIOXA* overexpression plasmid (Figure 3d) was first assembled and subsequently introduced into *Agrobacterium tumefaciens*. Agrobacterium-mediated infection then yielded a batch of wheat plants transiently overexpressing *TaMIOXA*.

Next, total RNA was extracted from the leaves of three independent *TaMIOXA* overexpression wheat lines and subjected to RT-qPCR analysis. As shown in Figure 3, the expression level of *TaMIOXA* was significantly upregulated in transgenic lines compared to the wild-type control group.

**Figure 4:**
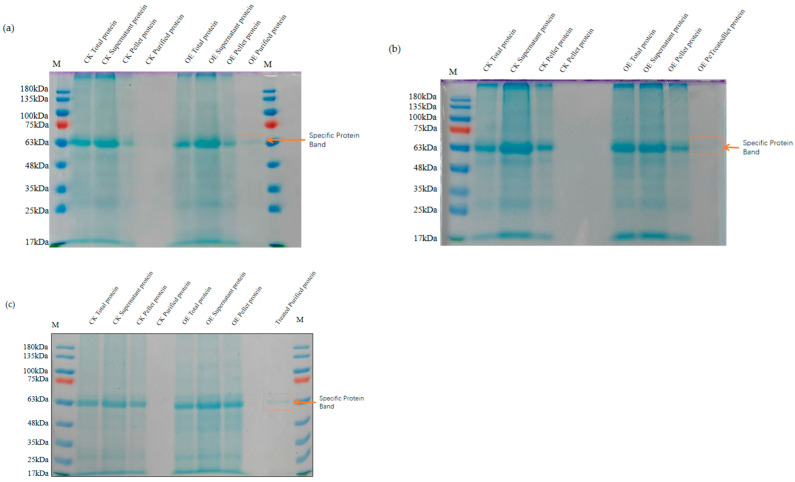
SDS-PAGE electrophoresis results for the extraction of wheat leaf protein. OE: *TaMIOXA* overexpression. (**a**) Bainong 207. (**b**) Yangmai 13. (**c**) Zhengmai 7698. The specific protein band for the His-tagged fusion protein was detected at 63 kDa.

Subsequently, leaf proteins from the three wheat genotypes were extracted and purified using a His tag affinity protocol. The purified proteins were then resolved using SDS-PAGE (Figure 4). A distinct band corresponding to the expected molecular weight (~61.7 kDa) was observed in the supernatant of *TaMIOX* overexpression lines, whereas no detectable band appeared in the purified fractions from the control plants. The HIS-tag purified proteins from the overexpression lines and the corresponding Western blot (WB) results are shown in Figure S2. These results confirm that the *pCAMBIA1302-TaMIOXA* construct is functionally expressed in wheat leaves.

### 2.4. Detection of Various Indicators of Overexpressing Strains

#### 2.4.1. Phenotype of Drought and High-Temperature Treatment

**Figure 5:**
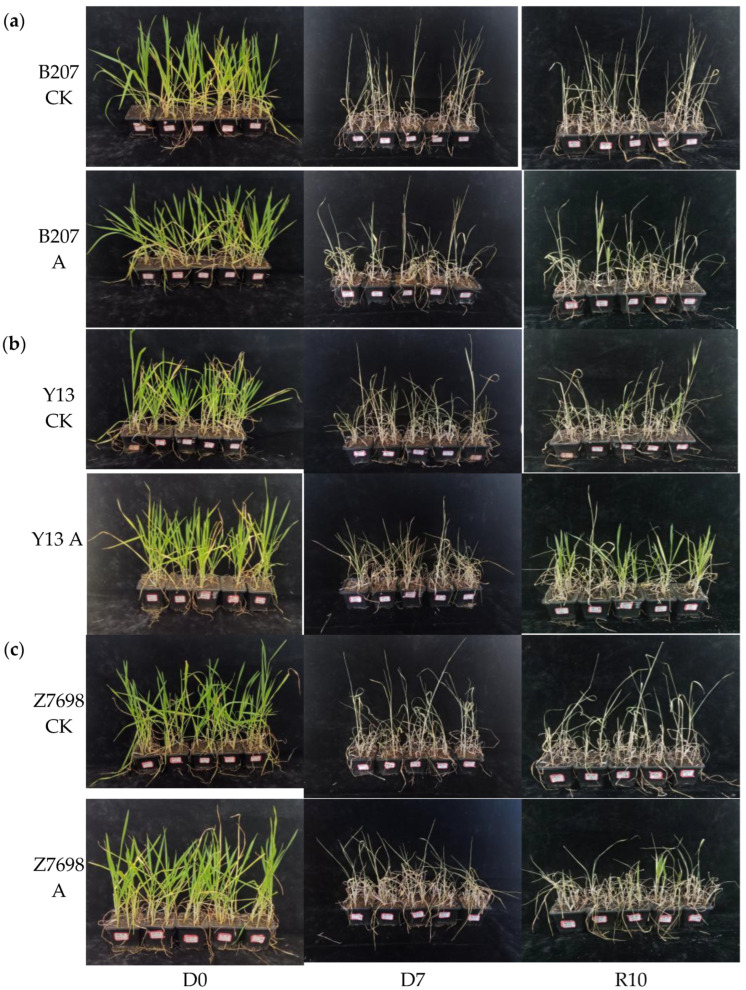
Images of control and overexpression groups subjected to dry-heat treatment for 0 days and 7 days, followed by 10 days of recovery from rehydration. (**a**) Bainong 207. (**b**) Yangmai 13. (**c**) Zhengmai 7698. D0 (Day 0); D7 (Day 7); R10 (Recovery Day 10).

After the initial characterization, the three wheat cultivars were grown under identical conditions for 73 days and then subjected to combined drought and high-temperature stress treatment for 7 days, followed by a 10-day recovery period with adequate irrigation (Figure 5). Survival rates were recorded and are summarized in Table 1. The *myo*-inositol oxygenase overexpression lines displayed markedly higher survival rates than their respective wild-type controls. Notably, the *TaMIOXA* overexpression line of Yangmai 13 achieved the highest survival rate (53.33%), representing a 40% increase relative to the control. Among the three cultivars, the *TaMIOXA* overexpression line of Zhengmai 7698 exhibited the most pronounced improvement in survival compared to its control group. The quantitative PCR analysis of the three wheat cultivars revealed that lines exhibiting high *TaMIOXA* expression displayed markedly enhanced tolerance to both drought and high-temperature stress. Among the tested varieties, the Zhengmai 7698 cultivar showed the most pronounced improvement in stress resistance.

#### 2.4.2. Detection of Physiological Indexes

**Figure 6:**
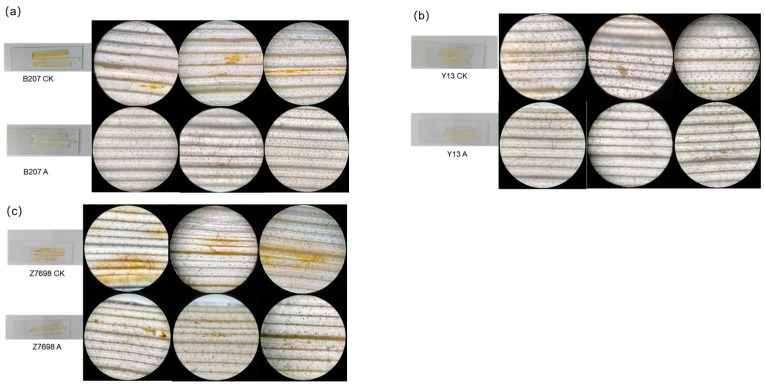
The control and overexpression groups were subjected to 24 h dry-heat treatment, after which the leaves were examined using DAB staining. (**a**) Bainong 207. (**b**) Yangmai 13. (**c**) Zhengmai 7698.

To further elucidate the role of *TaMIOXA* in the tolerance of wheat to high temperatures and drought, five plants were randomly selected from each treatment group after exposure to combined heat–drought stress. Through DAB staining observations [33], we found that the content of H_2_O_2_ in wheat leaves from the overexpression group was significantly lower than that of the control group after high-temperature and drought treatment (Figure 6). Among the tested wheat cultivars, Zhengmai 7698 displayed the greatest variation in staining, followed by Bainong 207, whereas the variation in Yangmai 13 was relatively unremarkable.

**Figure 7:**
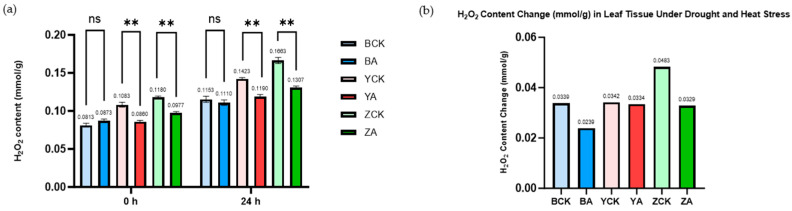
The control and *TaMIOXA*-overexpression groups were subjected to 24 h dry-heat treatment, after which the above-ground tissues of the wheat plants were harvested for hydrogen peroxide (H_2_O_2_) quantification. (**a**) H_2_O_2_ content in the three wheat cultivars (Bainong 207, Yangmai 13, and Zhengmai 7698) for both control and overexpression lines, measured before and after the 24 h dry-heat stress test. Statistical analyses were performed using two-way analysis of variance (ANOVA). ** *p* < 0.01; ns, not significant. Data are presented as mean ± SD; all experiments included n = 3 biological replicates. (**b**) A cumulative change in H_2_O_2_ levels (post-treatment minus pre-treatment) for the same three cultivars, comparing control and overexpression groups after the 24 h dry-heat exposure test.

The quantitative determination of H_2_O_2_ content before and after stress treatment corroborated the DAB-staining results. As shown in Figure 7, the H_2_O_2_ levels in all *TaMIOXA*-overexpression lines were significantly lower than the corresponding control plants. Specifically, the control plants Bainong 207, Yangmai 13, and Zhengmai 7698 exhibited H_2_O_2_ concentrations 1.04-, 1.19-, and 1.27-fold higher, respectively, than those of overexpression lines. Moreover, the relative accumulation of H_2_O_2_ (post-treatment versus pre-treatment) in the control groups was 1.42-fold, 1.02-fold, and 1.47-fold greater than that of the overexpression groups (Figure 7).

**Figure 8:**
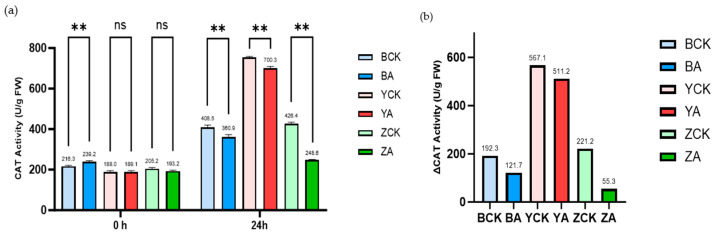
The control and *TaMIOXA*-overexpression groups were subjected to a 24 h dry-heat treatment, after which the above-ground tissues of the wheat plants were harvested for catalase (CAT) activity measurement. (**a**) CAT activity in the three wheat cultivars (Bainong 207, Yangmai 13, and Zhengmai 7698) for both control and overexpression lines, measured before and after the 24 h dry-heat stress. (**b**) Cumulative changes in CAT activity (post-treatment minus pre-treatment) for the same three cultivars, comparing control and overexpression. Statistical analyses were performed using two-way analysis of variance (ANOVA). ** *p* < 0.01; ns, not significant. Data are presented as mean ± SD; all experiments included n = 3 biological replicates.

We also measured the catalase (CAT) activity of wheat before and after a 1-day drought and high-temperature treatment (Figure 8). As shown in Figure 8, the CAT activities of all *TaMIOXA*-overexpression lines were lower than those of the corresponding control plants after the stress. Specifically, the CAT activities in the control groups of Bainong 207, Yangmai 13, and Zhengmai 7698 were 1.13-, 1.08-, and 1.72-fold higher, respectively, than in the overexpression lines. Moreover, the increase in CAT activity from pre- to post-treatment was markedly greater in the control plants. The fold increase in the control groups of Bainong 207, Yangmai 13, and Zhengmai 7698 was 1.58-, 1.11-, and 4.00-fold higher.

**Figure 9:**
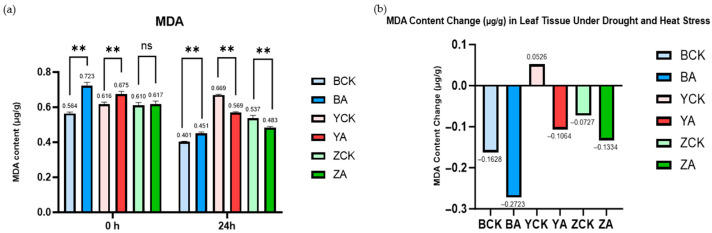
The control and *TaMIOXA*-overexpression groups were subjected to 24 h dry-heat treatment, after which the above-ground tissues of the wheat plants were harvested for malondialdehyde (MDA) measurement. (**a**) MDA content in the three wheat cultivars (Bainong 207, Yangmai 13, and Zhengmai 7698) was measured for both control and overexpression lines, before and after the 24 h dry-heat stress test. (**b**) Net change in MDA levels (post-treatment minus pre-treatment) for the same three cultivars, comparing control and overexpression groups after 24 h of dry-heat exposure. Statistical analyses were performed using two-way analysis of variance (ANOVA). ** *p* < 0.01; ns, not significant. Data are presented as mean ± SD; all experiments included n = 3 biological replicates.

As illustrated in Figure 9, panel (a) displays the malondialdehyde (MDA) content in both control and *TaMIOXA*-overexpressing groups across three wheat varieties before and after combined drought and heat stress treatment. Panel (b) presents the cumulative MDA content values before and after treatment. Here, we found an interesting phenomenon, normal in the face of abiotic stress in plants: MDA content will gradually increase, accelerating cell death. However, we performed a one-day dry-heat treatment on three wheat varieties. It was found that compared with the control group, the MDA accumulation in the Yangmai 13 overexpression group decreased more. The MDA reduction in the control plants of Bainong 207 and Zhengmai 7698 was 1.67- and 1.83-fold greater, respectively, than that observed in the overexpression lines. This shows that the increase in *MIOXA* expression will reduce the content of MDA in wheat.

**Figure 10:**
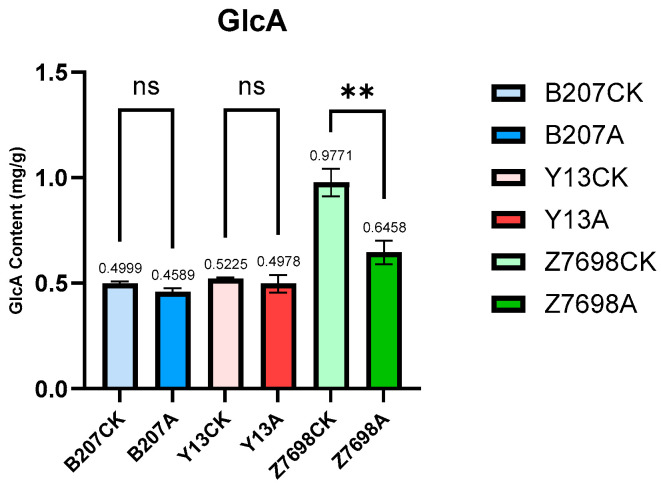
The glucuronic acid (GlcA) content was measured in the above-ground tissues of wheat from both the control and *TaMIOXA*-overexpression groups. Statistical analyses were performed using one-way analysis of variance (ANOVA). ** *p* < 0.01; ns, not significant. Data are presented as mean ± SD; all experiments included n = 3 biological replicates.

The detection results of glucuronic acid (GlcA) content in the control and *TaMIOXA*-overexpression groups (Figure 10) demonstrated that GlcA levels in all overexpression lines were lower than those in the corresponding controls. The reduction in GlcA in the control plants of Bainong 207, Yangmai 13, and Zhengmai 7698 was 1.09-, 1.05-, and 1.51-fold greater, respectively, than in the overexpression lines—an outcome that contradicts our initial hypothesis. Consequently, overexpression of *TaMIOXA* leads to a decrease in GlcA content.

**Figure 11:**
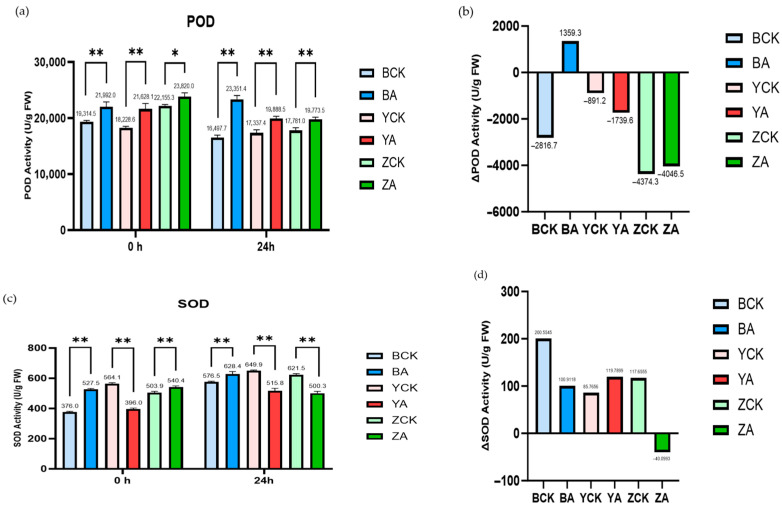
The control and *TaMIOXA*-overexpression groups were subjected to 24 h dry-heat treatment, after which the above-ground tissues of the wheat plants were harvested for peroxidase (POD) and superoxide dismutase (SOD) activity assays. (**a**) POD activity in the three wheat cultivars (Bainong 207, Yangmai 13, and Zhengmai 7698) for both control and overexpression lines, measured before and after the 24 h dry-heat stress test. (**b**) The net change in POD activity (post-treatment minus pre-treatment) for the same three cultivars, comparing control and overexpression groups, after 24 h dry-heat exposure. (**c**) SOD activity in the three wheat cultivars for control and overexpression lines, measured before and after the 24 h dry-heat treatment test. (**d**) The net change in SOD activity (post-treatment minus pre-treatment) for the three cultivars, comparing control and overexpression groups, after the 24 h dry-heat exposure test. Statistical analyses were performed using two-way analysis of variance (ANOVA). ** *p* < 0.01, * *p* < 0.05. Data are presented as mean ± SD; n = 3.

In addition, the detection results of POD (peroxidase) and SOD (superoxide dismutase) showed that (Figure 11) the enzyme activity of POD is not significantly affected by *TaMIOXA* overexpression. The results of SOD enzyme activity were similar to those of CAT enzyme activity, and the increase in enzyme activity in the overexpression group was significantly lower than that in the control group. This indicates that the reaction substrate of *TaMIOXA* may partially overlap with the substrate of SOD, but the specific type of superoxide consumed by oxygenase requires further investigation.

## 3. Discussion

We isolated the *TaMIOXA* gene from four wheat cultivars—Chinese Spring, Zhengmai 7698, Yangmai 13, and Bainong 207. Compared with the reference sequence, all four isolates shared an identical nucleotide substitution that resulted in a single amino-acid change, converting tyrosine to cysteine at position 185 (Y185C). This mutation likely represents a novel natural variant that arose through regional or environmental selection. In addition, we designed distinct primers (Table S1) for the PCR amplification of *TaMIOXB* (*Triticum aestivum myo*-inositol oxygenase B) and *TaMIOXD* (*Triticum aestivum myo*-inositol oxygenase D) based on the CDS of *TaMIOXA*, *TaMIOXB*, and *TaMIOXD*. Despite numerous attempts, neither allele could be amplified from any developmental stage of the four wheat varieties.

In this study, we generated transient *TaMIOXA*-overexpressing lines in three wheat cultivars and subjected them to heat and drought stress. Our results demonstrate that *TaMIOXA* overexpression markedly enhances wheat tolerance to both drought and high temperatures; this effect is especially pronounced in Zhengmai 7698, a principal wheat variety cultivated in Henan Province. This demonstrates that up-regulating *TaMIOXA* expression offers a novel strategy for mitigating wheat yield losses caused by recent dry and hot wind events under increasingly extreme high-temperature and drought conditions. *TaMIOXA* can be used as a screening marker for new drought- and high-temperature-resistant wheat varieties. However, for the same overexpression lines, the RT-qPCR results obtained the lowest overexpression for Yangmai 13, which may be related to the breeding screening. The primary cultivation region of Yangmai 13 lies south of the Bainong 207 and Zhengmai 7698 zones, where temperatures are comparatively higher. Targeted breeding has endowed Yangmai 13 with enhanced tolerance to elevated temperatures, and its baseline expression of *myo*-inositol oxygenase is relatively high. This elevated expression may also explain why the control group of Yangmai 13 exhibited the greatest survival rate after 7 days of combined drought and heat stress treatment. As such, *myo*-inositol oxygenase can serve as a novel screening marker for wheat varieties exhibiting high temperature and drought tolerance.

In addition, studies investigating which oxidant is consumed by plant *Myo*-inositol oxygenase during its catalytic activity are scarce. The prevailing view is that plant *Myo*-inositol oxygenase functions as an oxidase that utilizes molecular oxygen. Other radioisotope-labeling studies have demonstrated that, after ^18^O_2_ labeling, a portion of the oxygen atoms incorporated into the reaction product of glucuronic acid originates from molecular oxygen [34].

In this study, simultaneous monitoring of multiple wheat parameters during treatment allowed us to propose novel hypotheses concerning the oxidative substrates and stress-resistance mechanisms involved in the *TaMIOXA* reaction. After 1 day of treatment, the H_2_O_2_ levels in all overexpression groups were significantly lower than those in the control group. The cumulative amount of H_2_O_2_ in the overexpression groups was also comparatively reduced. On the contrary, the CAT enzyme activity of all overexpression wheat groups after drought and high-temperature treatment was lower than that of the control group. The level of CAT enzyme activity before and after treatment in the control group was significantly higher than that in the overexpression group. A comparison of cumulative H_2_O_2_ levels before and after wheat treatment indicates that the reduction in H_2_O_2_ accumulation observed in the overexpression lines is not attributable to catalase activity but is directly associated with the overexpression of *TaMIOXA*. *TaMIOXA* likely consumes H_2_O_2_ directly during the oxidation of *myo*-inositol.

**Figure 12:**
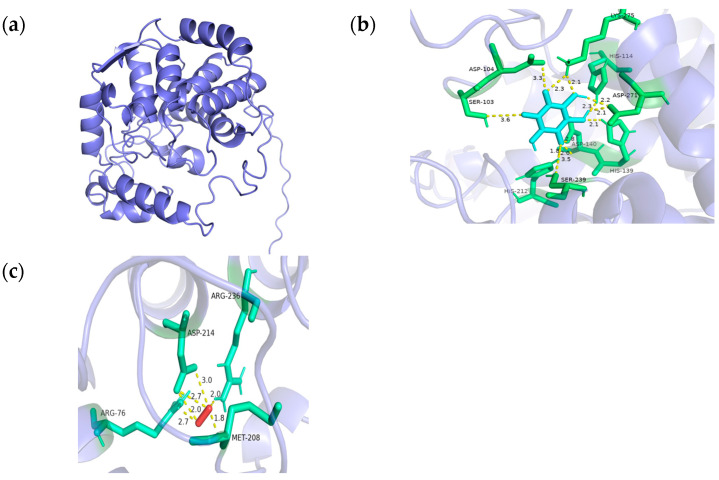
Molecular docking results of the TaMIOXA protein. (**a**) Protein modeling results of *myo*-inositol oxygenase generated by AlphaFold3. (**b**) The hydrogen bonding network from the docking of *myo*-inositol oxygenase with inositol. (**c**) The hydrogen bonding network from the docking of *myo*-inositol oxygenase with hydrogen peroxide.

We employed the AlphaFold3 online service to generate an ab initio model of *TaMIOXA* and subsequently performed molecular docking with AutoDock 4.26 to systematically screen and investigate the oxidation substrates and their corresponding binding sites on *TaMIOXA*. The results are displayed in a PyMOL map (Figure 12), and the corresponding hydrogen bond interaction network is marked. The mechanism of action and binding sites of *myo*-inositol oxygenase, inositol, and hydrogen peroxide were further predicted, as shown in Figure 12. The amino acids that have direct hydrogen bond interaction with inositol are serine (S103), aspartic acid oxygenase (D104), histidine (H139), aspartic acid (D140), histidine (H212), histidine (H238), serine (S239), aspartic acid (D271), and lysine (K275). Arginine (R76), methionine (M208), aspartic acid (D214), and arginine (R236) all experience direct hydrogen bonding interactions with hydrogen peroxide.

We performed a one-day dry-heat treatment test on three wheat cultivars. Apart from the control group of Yangmai 13, which showed an increase in MDA accumulation, the MDA content decreased in all other wheat groups. The increase in *TaMIOXA* expression during the early phase of treatment could have enhanced ASA synthesis while consuming a portion of MDA. Yangmai 13, the principal cultivar grown in southern regions, exhibits enhanced tolerance to high-temperature stress. Consequently, the expression level of *TaMIOXA* in the control group of Yangmai 13 was low, and the consumption (depletion) of malondialdehyde (MDA) was reduced. Therefore, we infer that *TaMIOXA* enhances wheat tolerance to high temperatures and drought, both due to the scavenging of reactive oxygen species and the promotion of ASA synthesis and other related pathways.

**Figure 13:**
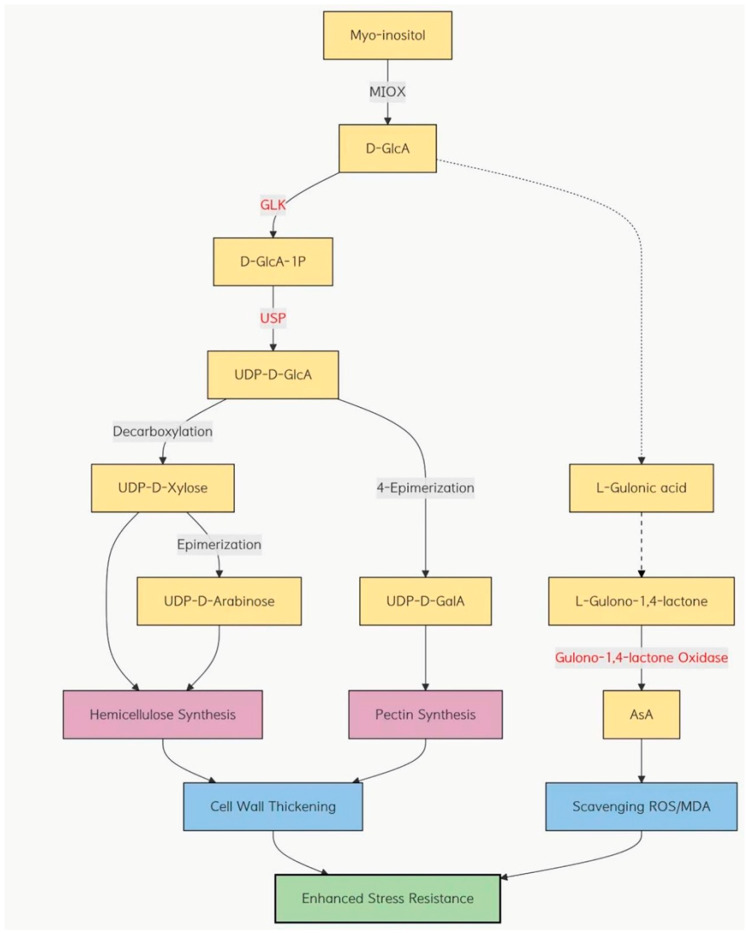
The *myo*-inositol oxygenase (*MIOX*) pathway and its hypothesized role in related pathways under combined drought and heat stress.

Finally, we measured the glucuronic acid content in both the control and overexpression groups of the three wheat varieties. The results demonstrated that the overexpression lines had a significantly lower glucuronic acid concentration than the controls, with the most pronounced decrease observed in the Zhengmai 7698 cultivar. Studies have demonstrated that overexpression of the *myo*-inositol oxygenase (*MIOX*) gene in *Arabidopsis thaliana* [35], tomato (*Solanum lycopersicum*) [36], and cucumber (*Cucumis sativus*) [37] markedly enhances the content of ascorbic acid (ASA). Combining the phenotypic observations and indices of three wheat cultivars after dry-heat treatment, we hypothesize that, during the early stages of abiotic stress, the expression of *myo*-inositol oxygenase is upregulated. This enzyme scavenges accumulated reactive oxygen species (ROS) and produces glucuronic acid. Subsequently, a portion of glucuronic acid may be converted into ascorbic acid (ASA) via the ASA pathway, thereby reducing malondialdehyde (MDA) levels and enhancing the stress tolerance of wheat (Figure 13).

## 4. Materials and Methods

### 4.1. Isolation and Cloning of TaMIOXA

Total RNA was extracted from wheat leaves using the total RNA extraction kit TRIpure Reagent (Heruibio, Fuzhou, China) [38], and then, RNA was reverse-transcribed into cDNA using HisScript II Q RT superMix for qPCR (Vazyme, Nanjing, China) [39]. The primers were designed based on the nucleotide sequence of TaMIOXA. The cDNA was amplified by polymerase chain reaction (PCR) using 2 × Es Taq MasterMix (Dye). The PCR product was then recovered using agarose gel electrophoresis and purified with the HiPure Gel Pure DNA Mini Kit (Magen, Guangzhou, China). The recovered products were submitted for sequencing; the resulting reads were aligned using SnapeGene^®^ 6.0.2 and subsequently translated into amino acid sequences [40].

### 4.2. Bioinformatics Analysis of TaMIOXA

The nucleotide sequence of *TaMIOXA* was translated into a protein sequence using SnapeGene^®^ 6.0.2 software. The protein structure of *TaMIOXA* was predicted using the AlphaFold Server (https://alphafoldserver.com/fold/5c40373bf5717a2e, accessed on 22 September 2025) provided by Google DeepMind. The platform is based on the AlphaFold3 model and can process various biomolecular inputs such as proteins and nucleic acids. The default parameters are used for prediction, and the custom template is not enabled. In order to perform molecular docking, this study obtained the three-dimensional structure files (format PDB) of hydrogen peroxide (H_2_O_2_) and superoxide anion (O^2−^) from the NCBI PubChem database (https://pubchem.ncbi.nlm.nih.gov/). The PubChem CIDs of the compounds were 784 and 5359597, respectively, and the structure of inositol was derived from ChemSpider (Inositol|C_6_H_12_O_6_) (https://www.chemspider.com/Chemical-Structure.10239179.html, accessed on 22 September 2025).

The translated amino acid sequences were analyzed using BLAST on NCBI platform, and the sequences with significant homology were selected for download [41]. Subsequently, phylogenetic tree analysis was performed using MEGA 7.0 [42]. After that, we used AutoDock 4.26 to perform molecular docking on the protein prediction structure of *TaMIOXA* [43], and the results were displayed using PyMOLWin 2.5.5 [44].

### 4.3. Expression Analysis of the TaMIOXA Gene

According to the method described by Wang et al. [45], the expression of *TaMIOXA* was evaluated via RT-qPCR. Customized RT-qPCR primers (Table S1) were produced by targeting the conserved sequences of 26S Ribosomal RNA gene [46]. For the RT-qPCR process, we used the SYBR Green Realtime PCR premix from TaKaRa in Beijing, China [47] to ensure accurate and reliable results. The relative expression of *TaMIOXA* was determined using the 2^−ΔΔCt^ method. The experiment was repeated using three techniques [48].

### 4.4. Preparation of Vector and Transformation of Wheat Plants

In this study, based on the coding-region sequence of the *TaMIOXA* gene and the sequence features of the *pCAMBIA1302-GFP* vector (Abcam, Hong Kong, China), *Nco*I and *Bgl*II were selected as restriction sites, and specific primers incorporating these sites were designed for homologous recombination experiments (Table S1). Subsequently, the constructed vector plasmid was transferred into *Agrobacterium tumefaciens* and transformed into wheat via *Agrobacterium tumefaciens* to obtain positive plants [49].

### 4.5. Plant Materials and Treatments

According to the planting conditions at different latitudes in the Huang-Huai wheat region, three wheat varieties were selected: Zhengmai 7698 (34°16′ N–36°22′ N), Yangmai 13 (29°–33° N), and Bainong 207 (31°23′ N–36°22′ N). From each variety, 150 seeds were chosen, primarily ovoid or elliptical, with intact morphology, a balanced seed-coat to endosperm ratio, and fine surface striations or hairs. After sterilization with hydrogen peroxide [50], the seeds were divided into two groups: a control group and a group subjected to *Agrobacterium*-mediated transformation. Following 2–3 days of germination, 50 healthy seeds from each treatment were transferred to cultivation pots, with 6–8 seeds per pot and 0.14 kg of dry soil per pot, which was then fully saturated with water. All treatment groups were maintained at 22 °C and 80 ± 5% relative humidity under a photoperiod of 16 h light/8 h dark and cultivated for 60–70 days. In each group, 30 healthy plants with complete roots, stems, and leaves were selected for stress exposure. After sampling, the samples were stored at −80 °C for further analysis.

### 4.6. Analysis of Physiological Indicators After Stress Overexpression of TaMIOXA in Wheat

Wheat plants in the overexpression groups (B207 A, Y13 A, Z7698 A) and the control groups (B207 CK, Y13 CK, Z7698 CK) were cultured in a light incubator for 10 weeks at 22 °C and 85% relative humidity, under a 16 h light/8 h dark photoperiod. The overexpression and control groups were simultaneously subjected to drought and high-temperature stress for 7 days, with conditions of 40 °C and 50% relative humidity for 14 h during the day, and 25 °C and 50% relative humidity for 8 h at night. After drought and high-temperature stress, the plants were re-watered and allowed to recover for 10 days. The above-ground parts of wheat were harvested, rapidly frozen in liquid nitrogen, and then stored at −80 °C for subsequent experiments. We quantified malondialdehyde (MDA) using the thiobarbituric acid (TBA) assay [6]. Hydrogen peroxide content was measured using the ammonium molybdate colorimetric method [51]; glucuronic acid was determined with high-performance liquid chromatography (HPLC); catalase (CAT) activity was assessed via UV spectrophotometry [11]; peroxidase (POD) activity was assessed using the guaiacol oxidation assay [52]; and superoxide dismutase (SOD) activity was assessed using the nitroblue tetrazolium (NBT) reduction assay [53], respectively.

## 5. Conclusions

In summary, wheat plants were subjected to combined drought and heat stress, which induced a significant upregulation of *myo*-inositol oxygenase transcripts. To investigate the role of *myo*-inositol oxygenase in wheat under abiotic stress, we generated three *TaMIOXA*-overexpressing wheat lines. Following exposure to high-temperature and drought treatments, the results demonstrated that *TaMIOXA* overexpression markedly enhanced the tolerance of wheat to both heat and drought stresses. Analysis of various physiological indices indicated that *myo*-inositol oxygenase enhances the plant’s stress resistance via the coordinated regulation of multiple pathways.

Under abiotic stress conditions, wheat plants accumulate reactive oxygen species (ROS), which upregulate the expression of *myo*-inositol oxygenase. This enzyme subsequently utilizes ROS (such as hydrogen peroxide) and *myo*-inositol to produce glucuronic acid [54], a process that may activate the ASA biosynthetic pathway, ultimately leading to ASA accumulation. ASA, in conjunction with superoxide dismutase (SOD), functions synergistically to scavenge malondialdehyde (MDA) in plants [55]. This cascade of reactions contributes to the maintenance of physiological homeostasis and improves abiotic stress tolerance. Thus, *myo*-inositol oxygenase represents a promising genetic target for enhancing heat and drought resistance in wheat, offering a novel strategy to mitigate the impact of extreme high-temperature events associated with global warming.

## Figures and Tables

**Table 1:** The recovery of wheat under stress conditions.

		Survival Count (Plants)	Initial Count (Plants)	Survival Rate (%)
CK	B207 CK	2	30	6.67
	Y13 CK	4	30	13.33
	Z7698 CK	0	30	0
TaMIOXA overexpression	B207 A	5	30	16.67
	Y13 A	16	30	53.33
	Z7698 A	12	30	40

## Data Availability

Statement: The original contributions presented in this study are included in the article/Supplementary Materials. Further inquiries can be directed to the corresponding author(s).

## Supplementary Materials

The supporting information can be downloaded at: https://www.mdpi.com/article/10.3390/ijms262210894/s1.

## Abbreviations

*TaMIOXA*	*myo-*inositol oxygenase gene in *Triticum aestivum* A-genome (*Triticum urartu*)
*TaMIOXB*	*myo-*inositol oxygenase gene in *Triticum aestivum* B-genome (*Aegilops speltoides*)
*TaMIOX* *D*	*myo-*inositol oxygenase gene in *Triticum aestivum* D-genome (*Aegilops tauschii*)
B207 A	*TaMIOXA* overexpressing lines of wheat cultivar Bainong 207
Y13 A	*TaMIOXA* overexpressing lines of wheat cultivar Yangmai 13
Z7698 A	*TaMIOXA* overexpressing lines of wheat cultivar Zhengmai 7698
B207 CK	control line of wheat cultivar Bainong 207
Y13 CK	control line of wheat cultivar Yangmai 13
Z7698 CK	control line of wheat cultivar Zhengmai 7698
CAT	catalase
SOD	superoxide dismutase
POD	peroxidase
GlcA	glucuronic acid
ASA	ascorbic acid
MDA	malondialdehyde
