# Supplementary material for: Overexpression of Myo-Inositol Oxygenase (TaMIOXA) Enhances the Drought and High-Temperature Resistance of Triticum aestivum L
Source: Int J Mol Sci. 2025 Nov 10;26(22):10894. doi: 10.3390/ijms262210894 (PMC12652114; doi:10.3390/ijms262210894)
Supplement: Supplementary file 1 [file ijms-26-10894-s001.zip › ijms-3932893-supplementary.pdf]

Supplementary Materials

```
TraesCS7A02G357800   ATGACCATCATCATCGAGCAGCCTCAGTTCGATGCGGCGGCGGAGAGGAAGGTCGCCGGCGACCCGGCGGAGCTCGTGCT    80
TaMIOXA              ATGACCATCATCATCGAGCAGCCTCAGTTCGATGCGGCGGCGGAGAGGAAGGTCGCCGGCGACCCGGCGGAGCTCGTGCT    80
Consensus            atgaccatcatcatcgagcagcctcagttcgatgcggcggcggagaggaaggtcgccggcgacccggcggagctcgtgct

TraesCS7A02G357800   CGACGGCGGCTTCACCGTACCGGACTCCAACGCCTTCGGCCACACCTTCAGGGACTACGACGCGGAGTCGGAGCGGAAGA   160
TaMIOXA              CGACGGCGGCTTCACCGTACCGGACTCCAACGCCTTCGGCCACACCTTCAGGGACTACGACGCGGAGTCGGAGCGGAAGA   160
Consensus            cgacggcggcttcaccgtaccggactccaacgccttcggccacaccttcagggactacgacgcggagtcggagcggaaga

TraesCS7A02G357800   AGACTGTGGAGGAGTTCTACCGCGTGAACCACATCAACCAGACGTACGAGTTTGTGCAGCGGATGCGGGACGCATACGGG   240
TaMIOXA              AGACTGTGGAGGAGTTCTACCGCGTGAACCACATCAACCAGACGTACGAGTTTGTGCAGCGGATGCGGGACGCATACGGG   240
Consensus            agactgtggaggagttctaccgcgtgaaccacatcaaccagacgtacgagtttgtgcagcggatgcgggacgcatacggg

TraesCS7A02G357800   CGGCTGGACAAGACGGAGATGAGCATCTGGGAGTGCATCGAGCTTCTCAACGAGTTCATCGACGACAGCGACCCTGACCT   320
TaMIOXA              CGGCTGGACAAGACGGAGATGAGCATCTGGGAGTGCATCGAGCTTCTCAACGAGTTCATCGACGACAGCGACCCTGACCT   320
Consensus            cggctggacaagacggagatgagcatctgggagtgcatcgagcttctcaacgagttcatcgacgacagcgaccctgacct

TraesCS7A02G357800   GGACATGCCGCAGATCGAGCACCTCCTCCAGACCGCCGAGGCCATCCGCAAGGACTACCCCGATGAGGACTGGCTCCACC   400
TaMIOXA              GGACATGCCGCAGATCGAGCACCTCCTCCAGACCGCCGAGGCCATCCGCAAGGACTACCCCGATGAGGACTGGCTCCACC   400
Consensus            ggacatgccgcagatcgagcacctcctccagaccgccgaggccatccgcaaggactaccccgatgaggactggctccacc

TraesCS7A02G357800   TCACTGGCCTCATCCATGATCTGGGCAAGGTGCTGCTGCATCCCAGCTTCGGGGAGCTTCCTCAGTGGGCAGTCGTAGGT   480
TaMIOXA              TCACTGGCCTCATCCATGATCTGGGCAAGGTGCTGCTGCATCCCAGCTTCGGGGAGCTTCCTCAGTGGGCAGTCGTAGGT   480
Consensus            tcactggcctcatccatgatctgggcaaggtgctgctgcatcccagcttcggggagcttcctcagtgggcagtcgtaggt

TraesCS7A02G357800   GACACCTTCCCCGTCGGCTGCGCGTTCGACGAATGCAACGTCCACTTCAAGTACTTCAAGGAGAACCCTGACTACCACAA   560
TaMIOXA              GACACCTTCCCCGTCGGCTGCGCGTTCGACGAATGCAACGTCCACTTCAAGTACTTCAAGGAGAACCCTGACTGCCACAA   560
Consensus            gacaccttccccgtcggctgcgcgttcgacgaatgcaacgtccacttcaagtacttcaaggagaaccctgact ccacaa

TraesCS7A02G357800   CCCGGAGTTCAACACCAAGTTCGGGGTCTACTCCGAGGGGTGCGGGCTGGACAACGTGCTCATGTCATGGGGCCATGACG   640
TaMIOXA              CCCGGAGTTCAACACCAAGTTCGGGGTCTACTCCGAGGGGTGCGGGCTGGACAACGTGCTCATGTCATGGGGCCATGACG   640
Consensus            cccggagttcaacaccaagttcggggtctactccgaggggtgcgggctggacaacgtgctcatgtcatggggccatgacg

TraesCS7A02G357800   ACTACATGTGCCTGGTTGCCAAGGAGAACAAGACCACCCTTCCTTCCGCAGGGCTGTTCATCATCAGATACCACTCCTTC   720
TaMIOXA              ACTACATGTGCCTGGTTGCCAAGGAGAACAAGACCACCCTTCCTTCCGCAGGGCTGTTCATCATCAGATACCACTCCTTC   720
Consensus            actacatgtgcctggttgccaaggagaacaagaccacccttccttccgcagggctgttcatcatcagataccactccttc

TraesCS7A02G357800   TACCCCCTGCACAAGCATGGAGCCTACATGCACCTGATGAACGAGGAGGACAAGGAGAACCTCAAATGGCTGCACGTCTT   800
TaMIOXA              TACCCCCTGCACAAGCATGGAGCCTACATGCACCTGATGAACGAGGAGGACAAGGAGAACCTCAAATGGCTGCACGTCTT   800
Consensus            taccccctgcacaagcatggagcctacatgcacctgatgaacgaggaggacaaggagaacctcaaatggctgcacgtctt

TraesCS7A02G357800   CAACAAGTATGACCTGTACAGCAAGAGCAGCGTCAGGATCGACGTCGAGGAAGTGAAGCCCTACTACATGTCGCTCATCG   880
TaMIOXA              CAACAAGTATGACCTGTACAGCAAGAGCAGCGTCAGGATCGACGTCGAGGAAGTGAAGCCCTACTACATGTCGCTCATCG   880
Consensus            caacaagtatgacctgtacagcaagagcagcgtcaggatcgacgtcgaggaagtgaagccctactacatgtcgctcatcg

TraesCS7A02G357800   ACAAGTACTTCCCGGGGAAGCTGCGATG                                                       908
TaMIOXA              ACAAGTACTTCCCGGGGAAGCTGCGATG                                                       908
Consensus            acaagtacttcccggggaagctgcgatg
```

Supplementary Figure S1. Sequence alignment between the reference TaMIOXA (TraesCS7A02G357800) and the PCR-amplified wheat sequence was performed using DNAMAN software.

Among them, TaMIOXB and TaMIOXD were attempted to be amplified from four wheat varieties representing different periods, but the target genes were not successfully obtained in either case.

Table S1. Primers used in this study.

Primer Name	Primer sequence (5' to 3')	Target Gene	Purpose
TaMIOXA-F	ATGACCATCATCATCGAGCAGC	TaMIOXA	**Cloning**
TaMIOXA-R	CCATCGCAGCTTCCCCGG	TaMIOXA	**Cloning**
TaMIOXA-1302-F	GGGACTCTTGACCATGGTACATGACCATCATC	TaMIOXA	Homologous Recombination
TaMIOXA-1302-R	TCTCCTTTACTAGTCAGATCTACCCATCGCAGCTT	TaMIOXA	Homologous Recombination
TaMIOXB-F	ATGACCATCATCATCGAGCAGC	TaMIOXB	**Cloning**
TaMIOXB-R	TCACCATCGCACCTTCCC	TaMIOXB	**Cloning**
TaMIOXD-F	ATGACCATCATCATCGAGCAGC	TaMIOXD	**Cloning**
TaMIOXD-R	TCACCATCTCAGCTTCCCCG	TaMIOXD	**Cloning**
TaMIOXB-F1	AGCATACCCATTACCGTCTTCTT	TaMIOXB	**Cloning**
TaMIOXB-R1	TCACCATCGCACCTTCCC	TaMIOXB	**Cloning**
TaMIOXD-F1	CAGACCCTGCTTCGCT	TaMIOXD	**Cloning**
TaMIOXD-R1	TCACCATCTCAGCTTCCCC	TaMIOXD	**Cloning**
TaMIOXD-F2	TTCGCTGCCGCGAG	TaMIOXD	**Cloning**
TaMIOXD-R2	TCACCATCTCAGCTTCCCC	TaMIOXD	**Cloning**
TaMIOXD-F3	TCTCAGACCCTGCTTCGCTG	TaMIOXD	**Cloning**
TaMIOXD-R3	CTTCGCTGCCGCGAGCTC	TaMIOXD	**Cloning**
Ta-MIOXA-QF	ATCGAGCAGCCTCAGTTCGA	TaMIOXA	**qPCR**
Ta-MIOXA-QF	CGCGGTAGAACTCCTCCACA	TaMIOXA	**qPCR**
26S-F	GCATATCAATAAGCGGAGGAAAAG	26S	**qPCR**
26S-R	GGTCCGTGTTTCAAGACGG	26S	**qPCR**

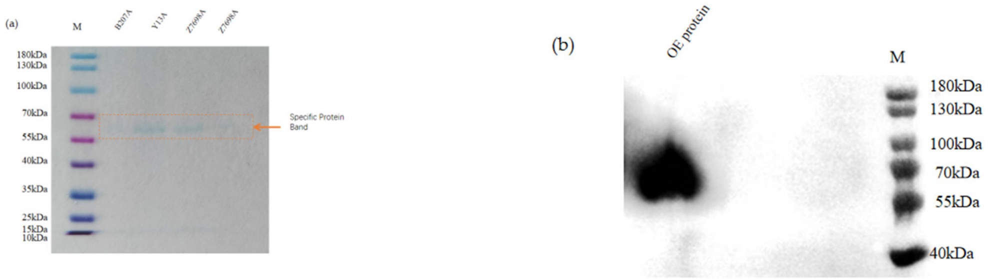

Figure S2. (a) SDS-PAGE analysis of leaf protein extracts from Bainong 207, Yangmai 13, and Zhengmai 7698 myo-inositol oxygenase A (MIOX A) overexpression lines after purification with His-tag affinity chromatography. (b) Western blot detection of MIOX A in leaf protein extracts from overexpression lines.
